# A Community-Based Cross-Sectional Study on Respiratory Health and Spirometry-Assessed Pulmonary Function Among Textile Workers in Tamil Nadu, India

**DOI:** 10.7759/cureus.55583

**Published:** 2024-03-05

**Authors:** Nirmal Sujitha I J, Sangeetha S, Yadukrishnan G, Priya M, Kirubhakaran Kanakaraju

**Affiliations:** 1 Community Medicine, Vinayaka Mission's Kirupananda Variyar Medical College Hospitals, Vinayaka Missions Research Foundation (Deemed to be University), Salem, IND; 2 Psychiatry, Believers Church Medical College Hospital, Thiruvalla, IND; 3 General Medicine, Vinayaka Mission's Kirupananda Variyar Medical College Hospitals, Vinayaka Missions Research Foundation (Deemed to be University), Salem, IND

**Keywords:** working environment, unorganized worker, spirometry, indoor air pollution, textile workers, pulmonary function

## Abstract

Introduction

The textile industry is one of the largest economic activities. Still, the laborers involved in it are exposed to various health-damaging air pollutants, putting them at risk of health issues including morbidities of the respiratory system. Therefore, this study aimed to assess the pulmonary function parameters of the workers involved in textile manufacturing-related jobs.

Methodology

A cross-sectional study was carried out among adult individuals who had been working in textile manufacturing-related jobs as their main work. The details such as sociodemographic, clinical, workplace conditions, and smoking habits were collected, as well as chest auscultation and lung spirometry using a hand-held spirometer. The participants who had normal and abnormal lung function patterns were statistically analyzed for potential influencing factors.

Results

The findings of the study conducted from 95 eligible participants identified that the pulmonary function parameters assessed by spirometry were in the abnormal range among 84 (88%) participants. Around 72 (82%) of them had a restrictive pattern, and six (6%) had both restrictive and obstructive (mixed) patterns of lung disease. Smokers and those who lacked cross-ventilation and/or fresh source of air in the workplace were more likely to have abnormal lung function. Participants who had their workplace and residence as same had significantly 6.44 (95% CI: 1.24, 33.36) times higher odds of having abnormal lung function in spirometry.

Conclusion

As workers involved in household-run textile manufacturing jobs are found to be at a higher risk of developing abnormal lung function, using personal protective equipment, following occupational safety measures, and improving the working environment to acceptable standards are essential to safeguard the respiratory health of laborers in such unorganized sectors.

## Introduction

The textile industry is one of the largest economic activities in India, meeting one of the basic human needs [1]. It mainly involves unorganized sector laborers engaged in various activities of the textile manufacturing processes such as handling, sizing, bleaching, dyeing, drying, curing, weaving, spinning, printing, and finishing of cotton-like natural or other synthetic raw materials [2,3]. All these sections of manufacturing processes emit particulates, volatile organic components, and other health-damaging air pollutants that are small particles that penetrate deep into the lungs, inflame the airways, enter the bloodstream, reduce the oxygen-carrying capacity of the blood, impair immune system response, and generate reactive oxygen species (ROS) [4-6]. The textile workers are significantly exposed to such oxidative stresses, making the textile industry plagued by air pollution problems [2,7]. The workers are vulnerable to consequent respiratory problems, pollutant-induced cardiovascular events, and other health issues such as position-related musculoskeletal problems, skin allergies, and noise-induced hearing disorders [8,9]. The World Health Organization (WHO) fact sheet attributed household air pollution to play a huge role in eventuating premature deaths and loss of healthy life years, with the largest burden in low- and middle-income countries where respiratory morbidity is one of the major causes [4]. Lakhs of Indian households are engaged in weaving and its allied occupations of fabric manufacturing, having their work setup inside their house under the same roof and being exposed to the dust generated around the clock [10]. The indoor environment and air quality standards are expected to be poorer in such households, and its members are in danger of all types of air pollution hazards. In India, there are government-laid directive principles to ensure safe and healthy working conditions for those employed in various economic activities in registered and licensed premises, which would perhaps not be applied to household-run economic activities [11]. The recently revised National Programme for Prevention and Control of Non-Communicable Diseases (NPNCD) in India has also identified air pollution as a high-risk factor for non-communicable diseases [12]. As respiratory morbidity is notably a major health hazard of textile manufacturing-related activities, this study aimed to assess the pulmonary function parameters of the workers involved in such jobs [9].

## Materials and methods

A cross-sectional study was carried out in the field practice area of a primary health center affiliated with a private medical college in Tamil Nadu, India. This is a semi-urban locality comprised more than half of the adult members involved in textile manufacturing-related jobs such as weaving and spinning, and many have their workplace and residence as same. This study included adult individuals aged 18 years and above who had been working in textile manufacturing-related jobs for at least six months a year. Those who were part-time involved in textile-related work were excluded from the study. The sample size was calculated using OpenEpi online software taking reference from another study that reported the proportion (p) of abnormal spirometry among the cotton mill workers as 70%, relative precision (E) as 20%, and design effect (DE) as 2, at a 5% significance level [13]. The required sample size (n) was calculated to be 92 using the following formula: n = p x q x (Z_α/2_ / E)^2^ x DE. The study proceeded with approval from the Institutional Ethics Committee (IEC) (VMKVMC&H/IEC/22/25). The eligible participants were identified from the above-mentioned study setting and recruited on their willingness by convenience sampling method. With informed consent, a structured questionnaire was used to collect details such as background information, workplace environment, duration of work, type of fabric worked on, smoking habit, respiratory diseases (such as tuberculosis, asthma, and COVID-19), any respiratory symptomatic manifestations, and comorbidities (such as diabetes and hypertension), and those data were meticulously entered into Google Forms. Chest auscultation was performed for all the participants by trained or trainee physicians, looking for any abnormal respiratory sounds. Pulmonary function parameters were determined using the CONTEC hand-held SP10 spirometer equipment (Contec Medical Systems Co., Ltd, Qinhuangdao (Hebei), China) that gives a visual display of the values as well as a computer-generated report when connected through the software from the same manufacturer. Qualified pulmonologist and physician from the internal medicine department guided on spirometry procedure and pulmonary function interpretation. Calibration was done by often testing the equipment on the same healthy volunteer and comparing the values for consistency. The spirometry maneuver was explained to the participants, and they were asked to perform it thrice. The best of three combined values of forced vital capacity (FVC) and forced expiratory volume in 1 second (FEV1) for each participant was taken for analysis. The Knudson reference equations were used for the interpretation of spirometry data [14]. The criteria for the diagnosis of obstructive, restrictive and mixed lung diseases was based on the algorithm by Johnson and Theurer [15]. All the data collected were exported to Microsoft Excel and checked for quality and correctness. Jamovi software v. 2.3.21 from the R core team was used for performing statistical analysis. The categorical data were summarized as proportions, and continuous measures were summarized as means and median values. Correlations for continuous measures were done appropriate to data distribution. The odds ratio and prevalence ratio were computed to establish association along with confidence interval and p-value at a 5% significance level. The sociodemographic, clinical, and work environment details were analyzed and compared among the participants who were found to have normal versus abnormal lung functions in spirometry.

## Results

The study was conducted over a two-year period, and information from 95 eligible participants was included in the study. The average and median ages of the participants were 45.7 and 45, respectively, of whom 63 (67%) were males. Out of the 71 participants who provided educational details, 12 (17%) were illiterate, 53 (75%) had some schooling, and six (8%) were college graduates. Out of all the participants, 58 (around 61%) were working in their own setup. The mean per capita income of all the participants was calculated to be Rs. 3,221 per month. The majority 83 (87.4%) were involved in the manufacturing of cotton fabric, 10 (10.5%) were involved in manufacturing polyester, and two (2.1%) were involved in silk manufacturing (Table 1).

**Table 1:** Background characteristics of the participants (N = 95)

Participants’ characteristics	Summary estimates (mean or median or percentage)
Age (years)	
Mean (SD)	45.7 (11.5)
Median (IQR)	45 (16.5)
Sex	
Males	63 (67%)
Females	32 (33%)
Duration of work (years)	
Mean (SD)	24.5 (12.1)
Median (IQR)	25 (15)
Per capita income (INR)	
Mean (SD)	3221 (2368)
Median (IQR)	2500 (2567)
Type of fabric	
Cotton	83 (87.4%)
Silk	2 (2.1%)
Polyester	10 (10.5%)

**Figure 1:**
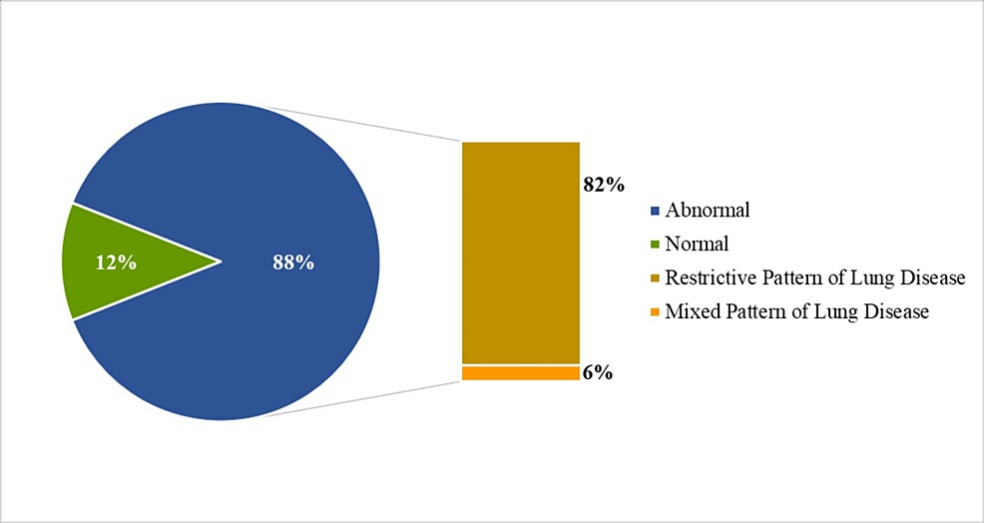
Distribution of participants’ spirometry-assessed lung function pattern

Pulmonary function parameters measured through spirometry were found to be abnormal in 84 participants, accounting for 88% (95% CI: 80% to 95%). Around 78 (82%) participants had a restrictive pattern of lung disease, while six (6%) had both restrictive and obstructive, i.e., mixed, patterns of lung disease. Only 11 (12%) had a normal lung function pattern in spirometry, and none of the participants had a purely obstructive pattern of lung disease (Figure 1).

Out of those who had a restrictive pattern of lung disease, over a third of them had it in severe form, i.e., FVC of less than 50%. The mean and median ages were comparatively higher for those who had a mixed pattern of lung disease. The lung function patterns were also significantly different among males and females, and no female was found to have normal lung function in spirometry. The work duration was also found to be higher for those who had a mixed pattern of lung disease. The predicted values of FEV1 and peak expiratory flow inversely correlated with the work duration.

Eleven of the study participants were smokers, and nine (82%) of them had restrictive lung disease. Among the study participants who lacked fresh source of air in the workplace, 20 (80%) of them had restrictive lung disease, and of those lacking cross-ventilation in the workplace, almost 24 (85%) had abnormal spirometry. Around 25 participants reported a positive history of diabetes mellitus and/or hypertension. Out of them, 19 (76%) had a restrictive pattern and three (12%) had a mixed pattern of lung diseases. Only one participant gave a history of tuberculosis and was found to have a restrictive pattern of lung disease, and another participant reported a history of asthma and had a mixed pattern of lung disease (Table 2).

**Table 2:** Lung function pattern related to certain sociodemographic, occupational, and clinical characteristics *For continuous measures, significance was assessed using the one-way ANOVA Kruskal-Wallis method, and for categorical data, significance was assessed using the chi-square goodness-of-fit method

Characteristics	Pattern of lung function			P-value*
	Normal	Restrictive	Mixed	
Age (years)				
Mean (SD)	45.9 (12.6)	45.3 (11.6)	50.8 (9.99)	0.415
Median (IQR)	47 (16.5)	44 (14.8)	51 (15)	-
Sex				
Males (n=63)	11 (17.5%)	48 (76.2%)	4 (6.3%)	0.041
Females (n=32)	0	30 (93.8%)	2 (6.3%)	
Work duration (years)				
Mean (SD)	25.4 (11.6)	23.7 (12)	33.7 (12.1)	0.181
Median (IQR)	25 (10)	22 (15)	32.5 (18.5)	-
History of smoking (n=11)	2 (18.2%)	9 (81.8%)	0	-
Lacking fresh source of air in the workplace (n=25)	5 (20%)	20 (80%)	0	-
Lacking cross-ventilation in the workplace (n=28)	4 (14.3%)	22 (78.6%)	2 (7.1%)	<0.00001
History of diabetes and/or hypertension (n=25)	3 (12%)	19 (76%)	3 (12%)	0.00004
History of tuberculosis (n=1)	0	100% (1)	0	-
History of asthma (n=1)	0	0	0.01% (1)	-
Respiratory symptomatic (n=10)	1 (10%)	8 (80%)	1 (10%)	0.0076
Abnormal respiratory sounds on auscultation (n=11)	0	11 (100%)	0	-

**Figure 2:**
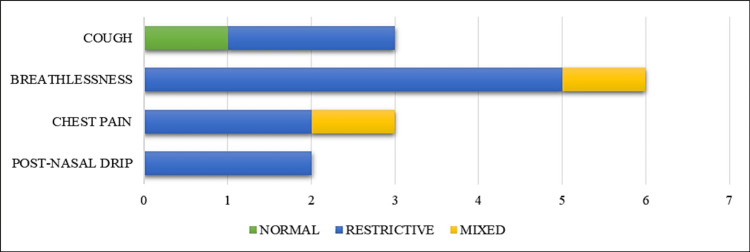
Respiratory symptoms among individuals with different lung function patterns in spirometry

A total of 10 participants reported having at least one or more respiratory symptoms such as cough, shortness of breath, chest pain, and/or post-nasal drip for at least one week or more. Out of those respiratory symptomatic, only one (10%) had normal spirometry, eight (80%) had a restrictive pattern, and one (10%) had a mixed pattern of lung disease (Table 2 and Figure 2).

During chest auscultation, abnormal respiratory sounds were observed only in 11 participants, all of whom had a restrictive pattern of lung disease in spirometry. The binomial univariate regression analysis revealed that those participants who had their workplace and residence as same had significantly 6.44 times higher odds (95% CI: 1.24 to 33.36; p = 0.026; Nagelkerke R^2^ = 0.144) of having abnormal lung function in spirometry compared to those having their workplace and residence at different places (Table 3).

**Table 3:** Having workplace and residence same as a predictor for abnormal lung function

Predictor	Estimate	P-value	Odds ratio	95% confidence interval	
				Lower	Upper
Workplace and residence same	1.86	0.026	6.44	1.24	33.36

The prevalence ratio was also significantly higher by 6.29 times (95% CI: 1.29 to 47.05; p = 0.02) to have abnormal lung function in spirometry for those participants who had their workplace and residence as same.

## Discussion

This cross-sectional study carried out among the main workers involved in textile manufacturing-related jobs revealed that 84 (88%) of the participants had abnormal lung function detected by hand-held spirometry. A study conducted in a cotton mill in Ahmedabad, India, where all the workers used reusable cotton cloth face masks during work time, reported that 70% of those workers had abnormal lung function in spirometry [13]. But, in the current study, the use of masks or any other personal protective equipment among the participants during work time was nil to negligible. Another study conducted in the Warangal district, India, also reported that all the pulmonary function parameters were significantly lower among handloom weavers [16]. The study conducted in Ahmedabad also reported a positive correlation between age and duration of exposure and highlighted that spirometric abnormality was more prevalent in those having a duration of exposure of more than five years [13]. In the current study, the mean age and work duration were the highest among those who had a mixed pattern of lung disease. It was also found that 87 (92%) participants had a work duration of more than five years, and, out of them, 77 (89%) had abnormal lung function in spirometry. Similar to the current study, another study conducted among handloom weavers in Nagpur, India, reported that the peak expiratory flow rate (PEFR) was significantly decreasing with increasing duration of exposure and also reported that respiratory morbidity was the most common health problem observed among the weavers [17]. The study report from the one conducted in Warangal also mentioned that 14% of the handloom weavers gave a history of bronchial asthma and around 9% of the people gave a history of allergic rhinitis, where cough and breathlessness were the predominant symptoms among the participants [16]. This is consistent with the current study and the study from Ahmedabad, where cough and breathlessness were the predominant symptoms among individuals with abnormal lung function in spirometry [13]. According to the present study, among those participants with habitual smoking habits, the majority had abnormal lung function. The study from Warangal also reported that all the pulmonary function test parameters showed lower values among smokers than non-smokers [16]. The study conducted in Nagpur also showed similar results that smokers had comparatively low PEFR values compared to past and non-smokers [17]. The current study unearthed the fact that those participants who live and work in the same place were at a higher significant risk of having deranged lung parameters, which might be because of their compromised poor housing and indoor environmental conditions. A study conducted among the textile workers of Rajasthan, India, also correlated the participants’ overall morbidity with environmental factors and housing conditions [9].

The current study is one of the very few studies that determined lung function among textile workers at the community level. The data collection along with the spirometry procedure and chest auscultation was carried out at the workplace of the participants by trained cum trainee physicians, which is one of the major strengths of the study. However, in the limitation part explaining the spirometry procedure to the participants and getting them done especially among the female participants was challenging, which would have introduced measurement bias overestimating the outcome. Few other limitations include lacking a control group from other occupations, as well as a certain number of missing values, which were also crucial to the study outcome. Nevertheless, the current study brought to light the respiratory health hazards of poor indoor working conditions and recommends using personal protective equipment during work time, improving the working environment to meet acceptable standards, providing health education toward following occupational safety measures, and avoiding unhealthy lifestyle habits and seeking appropriate health advice. Properly investigating the patient's occupational history is equally important from a healthcare service perspective [7]. The government laid regulations should also have a considerable focus on the unorganized sector of laborers who have household-run textile manufacturing or other economic activities. More research studies are needed that directly measure the indoor air quality of such dwellings in comparison with WHO standards attributing to the non-communicable disease burden among the inhabitants.

## Conclusions

The current study revealed that the pulmonary function parameters were abnormal in 88% (95% CI: 80% to 95%) of the workers involved in textile manufacturing-related jobs. Smokers, those participants who lack cross ventilation, those who lack fresh sources of air in the workplace, and those who live and work in the same place were more likely to have abnormal lung function. Henceforth, there is a dire and desperate need to emphasize using personal protective equipment, following occupational safety measures, and improving the working environment to meet acceptable standards for them. It is the duty of government and concerned national and international federations to have a considerable special focus on such unorganized sectors of laborers who have household-run textile manufacturing or other economic activities.
